# Quality of Life Philosophy IV. The Brain and Consciousness

**DOI:** 10.1100/tsw.2003.104

**Published:** 2003-12-01

**Authors:** Søren Ventegodt, Niels Jørgen Andersen, Joav Merrick

**Affiliations:** The quality of Life Research Center, Copenhagen, Denmark

**Keywords:** quality of life, QOL, philosophy, human development, holistic medicine, consciousness, brain, public health, Denmark

## Abstract

In this article we look at the brain’s structure and function from a philosophical perspective. Although the brain at micro-level, with its trillions of ultra-thin nerve fibers, is one of the most complicated structures in the known universe, you can still grasp its composition if you go up to the level of the cell. How this structure functions is not quite clear. You can understand its function at fiber level, because it is fairly simple, and you can understand it at cell level, but it is already vague. Roughly speaking, you can envision a single nerve cell as a tiny, independent computer whose behavior is dependent on continuous calculations of all input. At organ level, the function can be understood as an extremely complex pattern machine. Finally, the brain’s function can be understood at the cognitive level as what provides consciousness through its ability to keep order in our complicated reality. The superior function of the brain is to connect the real us, our higher self, to the surrounding world.The brain has been developed so that it can create all possible complex patterns. The connectivity seems to imply that the patterns of the human brain are 1000-dimensional. It is our vision that these complicated patterns arise from basic patterns in the quantum matter of which everything is created. In our opinion, our consciousness’ special utilization of a patterned aspect of nature is what lies behind inscrutable statements like “Man is created in God’s image”. We suggest that these patterns in matter are the basic, creative force that influences all living organisms. Unfortunately, science has only just begun to understand these patterns.The Bible’s description of the origin of man is two people eating from the Tree of Knowledge and as punishment they are expelled from the Garden of Eden. What does that mean? It means that, as conscious creatures, we no longer were an unproblematic, harmonious part of the world around us. The great question is why this consciousness about the world, provided by the brain, is not a gift that makes life better instead of getting us expelled from the Garden of Eden. We think that our real problem is the fact that we are still not in control of our consciousness. Instead of it serving us, we have become its slaves. If we come to understand brain and consciousness in order to solve this basic problem of our existence, we shall again be able to become a coherent part of the world, both as individuals and as a species. We share the vision that such an understanding of the problems of consciousness will make medical science holistic and will bring quality of life, health, and the ability to function to its patients.

## INTRODUCTION

The problem addressed in this paper is one of the most complex in biology and medicine and one that has been the subject for intense scientific exploration during the last 2 decades with many amazing new discoveries. There is a literature so vast that it can hardly be studied in detail by any researcher, and nobody seems to be able to read all the articles published in fine journals like *Behavioral and Brain Sciences.* A number of nice introductions, like Kandel and Schwartz' great book *Principles of Neural Science*[1], have been published during this period covering a number of aspects from philosophical to anatomical and physiological[1,2,3,4,5,6,7,8,9,10,11,12,13].

So what can we contribute to this huge body of knowledge on the brain and consciousness? Let us start with the very simple consideration that science is an interpretation of reality. When we describe the brain, we must make some initial choices about how to conceive mind, consciousness, understanding, and the project of science itself. So in this paper, we step back to look at the fundamentals. What do we know, from a philosophical and abstract perspective? What are the axioms we choose to believe in, so we can get on with the scientific exploration? In our opinion, this process of stepping back and looking at the greater picture is much too seldom done in our science, where we are so busy producing scientific papers full of models, theories, and data from measurements. It is so important in medical scientific research and of value to our patients that we know direction, reason, and the problems connected to consciousness, that we find it valuable to stop and think, abstractly and vaguely, about the connection between consciousness and brain[14]. We hope that the reader shares this opinion.

## THE BEGINNING OF THE ERA OF LARGE HUMANOID BRAINS

About 10,000 years ago something very strange happened here on Earth. Some rather primitive ape-men developed the ability to describe the world in a very detailed way using an abstract language. They developed mental consciousness. According to archaeological discoveries, the leap to modern man has been under way for about 100,000 years, starting with the mastery of fire and the use of very primitive tools. But not until a common description of the world appeared was it possible to work out social rules in tribal communities, develop a number of specialized techniques, and develop the very complicated townships, which appeared for the first time around then.

How may we understand the evolution of mankind and the emergence of consciousness? A good place to start is the study of the human brain, which is quite unique. How was the brain developed and what were the forces that spurred this development? It is our personal vision that complicated basic patterns in the quantum matter[8,15], of which everything is created, has served as a template, because the brain has been developed so that it can create all possible complex patterns, as we shall see. In our opinion, our special utilization of a patterned aspect of nature[16,17] is what lies behind inscrutable statements like "Man is created in God's image". As we see it, these patterns in matter are the basic, creative force that influences living organisms. Unfortunately, science has only just begun to understand these patterns[18,19,20].

We shall look at the brain's structure and function. Although the brain at micro-level, with its trillions of ultra-thin nerve fibers, is one of the most complicated structures in the known universe, you can still grasp its composition if you go up to the level of the cell[21,22,23,24,25]. How this structure functions is not quite clear. You can understand its function at fiber level, because it is fairly simple[1], and you can understand it at cell level, but it is already vague[26,27,28,29,30]. Roughly speaking, you can envision a single nerve cell as a tiny, independent computer whose behavior is dependent on continuous calculations of all input. At organ level, the function can be understood as an extremely complex pattern machine. Finally, the brain's function can be understood at the cognitive level as what provides consciousness through its ability to keep order in our complicated reality[7,31,32,33,34]. The superior function of the brain is to connect us to the surrounding world, at least for the brains of a size we can manage to study[35].

Apparently, the brain systematizes all our experiences and verbal inputs into that map of reality. This map is not just a static depiction; it contains time so that our plans and strategies for realizing our innermost dreams are also part of this map[14].

The Bible's description of the origin of man is two people eating from the Tree of Knowledge and as punishment they are expelled from the Garden of Eden. What does that mean? It means that, as conscious creatures, we no longer were an unproblematic, harmonious part of the world around us. We were no longer as one with the animals and plants of the Garden of Eden.

The great question is why this consciousness about the world provided by the brain is not a gift that makes life better instead of getting us expelled from Eden[36,37,38]. We think that our real problem is the fact that we are still not in control of our consciousness[39]. Instead of it serving us, we have become its slaves. If we solve this basic problem of our existence[40], we shall again be able to become a coherent part of the world, both as individuals[41,42] and as a species.

## THE BRAIN IS MYSTERIOUS

The brain is one of science's greatest puzzles. We have only just begun to understand how the brain is able to interpret data from the eyes and the ears, how the motor patterns that coordinate the body's scores of muscles are arranged during walking, how we can recognize a particular face among billions, and speak ten different languages with thousands and thousands of words, as some people are able[1]. And we have not even begun to understand what it means to understand. How is man able to design things like the theory of relativity, quantum mechanics, and mathematics?

Today there are many models that are vaguely starting to explain some of the things that take place in the brain's layer of nerve cells, e.g., "neural networks", and promising models of the creation of very complex patterns from the repetition of simple processes[19]. But really, it is not very impressive when you think of the creativity and efficiency of our brains. Without insight into matter itself and biology, we shall never be able to understand the basic principles behind the brain and consciousness.

Let us introduce the problem of the brain with a couple of examples of the mysterious connection between brain and consciousness.

It once happened that a university student with practically no brain was used in a brain-scan test[43]. When he was still a fetus, the main part of the cerebral substance had been replaced by water. Usually, this condition —hydrochephalus, with water in the head — leads to severe mental retardation, but this person managed to reach university level without obvious mental deficiencies. His brain consisted of an edge of tissue only millimeters or a few centimeters thick, lining the cranium. The traditional explanation claims that normally we only use 10% of the brain and therefore the student possessed exactly the amount that was needed. But if we observe the functions of a normal brain, we will see that every brain cell is working more or less constantly. If we could manage with 10%, we probably would not have developed our large brain. And still, there is something wrong, because apparently there are some who can manage with such a small brain.

An experiment with cats showed similar results[43]. During a test, a researcher surgically removed most of a cat's brain apart from the areas that covered vital reflexes. He had the cat walk around on the tables during a large conference with neurologists and people in brain research and challenged the assembly to correctly diagnose the cat. Nobody was able to guess that the cat had no brain, because it behaved almost normally.

It comes as something of a surprise that our knowledge of life is born by life itself, but if we remember everything that Hydra was able to do without a brain, it does not appear so shocking after all[4]. This is just meant as a warning against too quick and simple conclusions.

## THE STRUCTURE OF THE BRAIN

In our attempts to understand the brain and consciousness, let us enter the brain and see what takes place in there starting with the brain structure. Our main interest is the cerebrum cortex, which was especially developed in our transition from ape to man.

The human brain consists of a bisected cerebrum weighing approx. 1 kg and situated inside the head. The cerebral cortex consists of approx. 25,000,000,000 brain cells that constantly receive messages from thousands of the other cells, and they in turn communicate with thousands of other cells in an extremely complicated and ramified pattern.

There is an incredible proliferation of cerebral connections within the brain. Neural connections run from numerous cells to the other half of the brain or to distant parts within their own hemisphere. Therefore, it hardly seems likely that the brain is functionally bisected into two essentially different halves, which is the opinion of several neuropsychologists. Despite the anatomical bisection, it is actually a closely knit whole with only minor areas possessing well-defined and isolated functions.

All these brain cells are interconnected through an almost incredible mass of neural fibers, nearly 1,000,000 km, which would circumvent the Earth 25 times. The brain can contain all these impulses because they are only one thousandth of a millimeter thick.

## VIEW OF THE PHYSIOLOGY OF THE BRAIN

Neural impulses run from one nerve cell to another. Apart from a few details, the opinion today is that we fully understand the distribution of the neural signal from one nerve cell to the next.

The next level of understanding is the cell. The cell deals with incoming signals by adding them up one by one (the actual summary function is dependent on the individual cell and can be very complicated). Thus the cell can be seen as a computer that calculates the input received and forwards new signals when the result is correct. There is little doubt that the cell has such a summary function, but we are convinced that this function is continuously modified by the biological system through biological information.

At levels above the cell level, it becomes almost impossible to follow what happens in the brain, because the brain produces functional patterns of nearly infinite complexity, which are also modeled by input from life's information in a way that is not understood at all[44]. The effect, however, is that information is transferred from the depth of the organism to the brain and this is a decisive input to our dreams and intuitive awareness of the world. We imagine that brain patterns can be directly regulated by the organism's information system, as in dreaming. This is parallel to the way the self-organization of the cell's molecules is governed by the cell's biological information system[38].

Much research has been carried out in the individual subareas of the cerebrum cortex, especially the optical cortex, which is the area that interprets vision. The hope is to pick up how the brain is able to see and thus to produce artificial, electronic eyes for use in military surveillance, for example. Science has advanced somewhat towards analyzing how optical impressions are gradually led to higher and higher levels of complexity. Unfortunately, scientists lose the threads just where it starts to get interesting, when the optical impressions received from the retina are about to make sense[1].

A large number of areas with fixed functions are known, but most of the cerebrum cortex is integrative, that is, occupied with interpreting information received by the brain. The frontal lobe deals with the highest integrative levels, the top of the pyramid of consciousness, and keeps track of space, time, and abstract ideas[1].

## THE BRAIN IS NOT A COMPUTER

At some time in the future, a very fast computer (perhaps 1,000 times faster than the ones we know today) may be able to copy the ability of the human brain to organize the world. But the experience of meaning and consciousness only come from life itself. Therefore, a computer can never become conscious or understanding in an intelligent way the way a human being is.

Many scientists and other people try to reduce the brain and consciousness to something inorganic. Especially among physicists, the so-called "neural network" is a hot research subject. However, these fairly simple physical models only bear passing resemblance to the human brain. The dynamic we know as "thought" is not analogous to the lifeless physical models that are on the market today, however sophisticated they may be.

In his book *The User Illusion*[45], the author Tor Nørretranders has described the brain as a complicated calculator that more or less creates consciousness as a by-product of the process that reduces the complex to the simple (seemingly a misinterpretation of the Libet's famous experiments[46]). In our opinion this is a materialistic reduction of all those fantastic things that take place in the human brain. Such a description disregards the fact that the brain is a living organ in a living organism, and it does not do justice to consciousness at all. We hold the view that the foundation of consciousness and awareness stems from life itself, from the communication between all the cells of the organism, and not just from the brain. Therefore, the brain does not actually create consciousness; it just provides it with the particular quality or mental order we call reason.

The brain bridges the gap between the depth of life and the world around us; nerve cells communicate with each other and the other cells in the body in two ways. First, like any other cell, through an exchange of biological information of an unknown nature[14,37] which, at conception, provides the brain with its shape and structure[5]. Second, through chemical and electrical communication where electrical neural impulses can be transported along neural fibers and jump from cell to cell via chemical synapses. Hormone-like substances secreted by the brain itself that are called neuropeptides also chemically influence nerve cells.

In order to understand the function of the brain, both forms of communication — the one that disseminates biological information and the one that communicates chemical-electrical signals — are needed. This is because the brain bridges the gap between life inside the organism and the outside world[14]. The first form of communication, which may be called the basic biological communication that makes the brain an integrated organ in the organism, creates the bridge towards the inside. The bridge towards the outside, to the outside world, is built with the help of the other form of communication, the electrical and chemical, that is well suited for sensing via the sensory organs.

One might say that the brain receives the signals about our inner dreams and wishes (the biological potentials) through the former kind of communication, while the information about the outside world is received through the latter.

The brain's real business appears to be the production of electrical patterns that are so like the biological patterns (whose nature today is unknown but probably not electrical, more like quantum mechanical) that the description of reality can bridge the gap between the inside and the outside. In this way, meaning is created of all the input we receive from the outside through our sensory organs, a meaning, which, popularly speaking, occurs when sense perceptions from the outside agree with the organism's inner biological order.

What the brain is doing is organizing reality. It handles all sense perceptions and impressions that over time pass our way and reorganizes these sense perceptions into plans and strategies for the future in accordance with our inner dreams and wishes.

## THE ORDER OF OUR REASON AND INTUITION

The result of the brain's activity is the order of reason. The whole world has been neatly arranged and described to the smallest detail in time and space. Still, this order is somehow artificial, a reduction of an infinitely complicated reality into something that is easy for us to comprehend and relate to.

As organisms we are able directly to experience reality — in principle. We use our wholeness instead of our brain and senses. Such a direct experience ought to provide us with a much more dynamic and correct picture of reality. However, the picture is so chaotic and disorderly that it does not make much sense to our reason, which is practically drowning in the flood of information. It is this direct access to reality that we usually call intuition, a sure knowledge situated at a level that is lower than that of the model of the world, which is created by language and reason.

We possess two sources of knowledge. One is through the senses, where the impressions become a certain, well-organized picture of reality with the help of the brain's organizing process. All the things that we learn can be stored in a neat and well-organized way in this picture, which produces our map of reality. Usually, we call this organizing faculty reason.

Our other source of knowledge is intuition. Here awareness takes hold of the reality of life, both on the inside and outside, in a more direct but far less organized way. In this way, intuition is directly connected to the information system living matter uses. Through this information system, our intuition can draw upon all the information contained in the recipe for a human being, as well as all the knowledge life acquires through its intense dialog with other living beings at all the levels that make up the world.

People can function at his optimum only when both intuition and reason are cultivated and in balance, that is, when reason gives intuition ample space and when intuition does not cover subjects that are better handled by reason.

## THE MYSTERY OF AWARENESS

The nature of human awareness is mysterious. Let us distinguish between consciousness and awareness. To us, consciousness is a cultivated form of awareness. Consciousness is connected to the brain in the shape of reason, while awareness is connected to life itself. The awareness of the individual cells coalesces and makes up the raw material for the organism's overall awareness.

There are people who can relate how they witnessed operations performed on them although they were fully anesthetized. What makes it difficult to disregard such stories is the fact that what the patients purported to have experienced, while their brain was fully anesthetized, such as dialogs and other events, actually did take place. Something or other in their being was aware, even though the brain was anesthetized. Apparently in these cases man is aware without being conscious and without the senses being active.

We may think of awareness as a faculty that can grab hold of multitudinous things. When the cells' united awareness gets hold of the brain, it lets us experience the outside world through our senses and through the organized interpretation we call our map of reality, that is, our reason's consciousness of the world. This is different from the direct, spontaneous, and unreflective experience of the world that the brain receives when awareness grabs hold of reality directly and without consulting the brain and the senses. Awareness is the ability produced by being fully and completely present in your life.

## NIGHT DREAMS — A WINDOW OF THE SOUL

When we sleep, half the time is spent dreaming. Actually, we also sometimes dream when we are awake, but daydreams, imaginings, and other creative thought processes are of a more fragile quality, because our brain is already using most of its energy on maintaining the awareness of the world that is descriptive of the ordinary waking state.

But at night, when our senses are disconnected from the world, the brain is set free and is able to carry out the creative process we call dreaming. Probably this process is not qualitatively different from daydreaming or having visions about the good life, but night dreams are still of a different nature than daydreams. In our dreams we get much closer to our body, our life, and our organism. When we are disconnected from the outside world, we become temporarily free and are able to shape our pictures in the brain according to our profound desires and potentials.

The dreaming state can be registered with electrodes attached to the brain. We see that the brain works in different, characteristic rhythms (high-frequency alpha and beta waves and low-frequency delta and theta waves). During the night we have periods with very active dreams (with extremely high-frequency brain waves) and periods without dreams (with extremely low-frequency brain waves).

We think that what happens here is that awareness swings from being mostly in the brain to being mostly in the body when you go from dreaming to not dreaming. An interpretation of this can be that the natural course of sleep is about fusing brain and body and brings the map in harmony with the inner life just like the daily, outer activity is about bringing the map in harmony with the outside world. In this way, we try every night to heal ourselves and create the sound bridge between the depth of life and the outside world[14].

Dreams in the Freudian and Jungian tradition[47] are seen to be about our conflicts and existential problems. A lively debate occurs inside us when what we have learned about the world is inconsistent with life itself. Subconsciously we try to become whole, healthy, happy, and well-balanced people. Therefore, dreams hold an enormous potential for healing and curing. When we dream and really relax it seems that all parts of our organism are tied together a little better, as if disorders and breaches in our existence are healed. It is important to notice that this conception of dreams as a bridge to our soul or genuine self (higher self, wholeness) is not shared with all researchers in the cognitive traditions.

## THE WORLD IS MODELED ON THE PATTERNS OF THE BRAIN

To understand what happens in the brain, it should also be mentioned that a quarter of the energy of the human organism is spent on keeping the brain going 24 h a day. Nerve tissue has a formidably high metabolism. Every nerve cell is constantly sending signals to others, the brain as whole never rests.

Now, what is the brain doing with all these nerve fibers and all that energy? Apparently it produces very advanced patterns that are the templates we use for shaping our whole description of the world. To get an idea of just how complicated these patterns are, you can think of the patterns in water, where impulses are only moved from one molecule to the next in two or three dimensions. The surface of water, with its two dimensions, produces rings and the like. With three dimensions, you can see various types of whirls and spindrifts. The ramifications in the brain make the brain's patterns 1,000-dimensional.

The patterns are further complicated by the fact that each area of the brain (each nerve cell) communicates not only with its neighbor, but also with distant areas all over the brain that again react back to the first place via a number of intermediate steps. However, it does not become really complex until the brain starts working and keeps feeding every nerve cell with its own and other brain cells' output. Such iterative processes where the same simple process is repeated time and again in almost the same way have led to the most complex patterns (just think of the many levels of spirals and patterns of the Mandelbrot fractal, or whirls in the water or clouds in the sky[18,19]). So what happens when the processes in question are patterns in the brain's incredibly complicated network?

Then it no longer seems strange that our brain can model and create all the various shapes, phenomena, processes, and basic qualities of the outside world that we use for constructing our interpretation of reality. However, it is still shocking that our head is able to contain such an incredibly extensive and dynamic model of the whole world.

One of the real mysteries is memory — that all these impressions, experiences, and phenomena can be stored in the brain and recalled in an orderly way almost immediately. On the other hand, perhaps it is no stranger than the recipe for the human being that is stored in the biological material. The principle of storage is probably exactly the same. Life's ability to handle information is almost unfathomable.

## THE BRAIN CREATES ORDER THROUGH A HIERARCHY OF CONCEPTS

The brain organizes reality for the person and it creates order from a chaotic reality. The brain can organize every received element of reality into groups of elements that have common properties. In this way all the elements of reality are organized hierarchically, so that elements combine at still higher levels to finally produce our abstract concepts of the world. You could say that reality is arranged into a "pyramid of consciousness" with everything concrete forming the broad foundation and the few, extensive concepts at the top of the pyramid.

Everything we know is organized according to properties. Some examples are kitchen utensils, others vehicles. The kitchen utensils can be subdivided into pots and pans, while the means of transportation can be subdivided into cars, bicycles, etc. In the same way all the processes that exist in reality are categorized as being creative, destructive, for maintenance, or for transformation. All phenomena can be grouped according to certain specific qualities like shape and color, or according to abstract qualities such as value, meaning, structure, etc.

The whole of reality is organized into main areas through concepts such as "matter" (the physical, chemical, or inorganic, dead), "life" (the organic), "consciousness" (the psychological, ethical, philosophical, and religious), and "society" (economy, law, and politics) as well as a number of other concepts. This order is the leading principle in what we call reason, but the actual structure behind this order, the system in which reason acts, is still not understood, neither philosophically nor scientifically.

This profusion of concrete phenomena and processes that characterize our world is gathered into an ordered description of the world through abstract concepts and principles. When you add it all up, it becomes the map of reality. This map is constructed through an ingenious combination of the brain's ability to create order in the world with the help of self-organizing patterns and life's ability to add meaning to such patterns.

## CONCLUSION: THE BRAIN AND REALITY

Thus our mind contains a description of the world that is very complicated and that is made up of endless numbers of these extremely complicated patterns that are added together in order to produce faces, flowers, and trees and even products such as metal surfaces.

Everything that we have experienced in life, all the pencils we have used and the glasses of water we have lifted, has been boiled down into a very complicated and dynamic description of the reality contained in our brains. We just have to see the reflection of the sun in a glass to acknowledge that we are facing a water tumbler. How can this be? It is because we have seen so many glasses that now we master visual perception almost completely.

What makes our description of the world really effective is that we are able to sprinkle our personal history, all our previous experiences and interpretations, over every new environment or locality that we enter. Thus every sensation is tinged with those like it that we have seen before, and almost any scene immediately becomes a whole and meaningful picture with people, furniture, trees, or whatever. This is very practical and you do not really need to spend much energy on what you are actually seeing, because you are able get a sense of it very well purely by reflex. You only need a few sensory clues and everything you have learned and experienced about the world becomes present again.

Now we are able to understand what is the evolutionary purpose of the brain: To create order out of the chaos of reality. With a sound model of the world, we become incredibly efficient at perceiving, ordering, and acting in the world. Thus, through this ability man has become the master of matter and has been able to invent zippers and space shuttles. The problem is that, concurrent with mastering matter, we have lost our grip of our soul[48,49,50,51]. We have lost our way in the mental maps of the world[44,52].

If we come to understand brain and consciousness and solve this basic problem of our existence, we shall again be able to become a coherent part of the world, both as individuals and as a species. We share the vision that such an understanding of the problems of consciousness will make medical science holistic and will bring quality of life, health, and the ability to function to its patients.

## ACKNOWLEDGMENTS

This study was supported by grants from The 1991 Pharmacy Foundation, as well as by supplementary grants from Goodwill-fonden, the JL-Foundation, E. Danielsen and Wife's Foundation, Emmerick Meyer's Trust, the Frimodt-Heineken Foundation, the Hede Nielsen Family Foundation, Petrus Andersens Fond, Wholesaler C.P. Frederiksens Study Trust, Else & Mogens Wedell-Wedellsborg's Foundation and IMK Almene Fond. We gratefully acknowledge the critical scrutiny and expert linguistic assistance of Ib Ravn, Ph.D. and also the assistance of the philosopher Maximilian Kroman. The research was approved by the Copenhagen Scientific Ethical Committee under number (KF)V.100.2123/91.

## BIOSKETCHES

**Søren Ventegodt, MD,** is the Director of the Quality of Life Research Center in Copenhagen, Denmark. He is also responsible for a Research Clinic for Holistic Medicine in Copenhagen and is a popular speaker throughout Scandinavia. He has published numerous scientific or popular articles and a number of books on holistic medicine, quality of life, and quality of working life. His most important scientific contributions are the comprehensive SEQOL questionnaire, the very short QoL5 questionnaire, the integrated QOL theory, the holistic process theory, the life mission theory, and the Danish Quality of Life Research Survey, 1991–94 in cooperation with the University Hospital of Copenhagen and the late pediatric professor Bengt Zachau-Christiansen. E-mail: ventegodt@livskvalitet.org. Website: www.livskvalitet.org/

**Niels Jørgen Andersen, MSc,** Professor, Department of Innovation and Economic Organization, Norwegian School of Management. This department conducts research and provides teaching in central topics related to innovation, business development, management of global companies, business history, and economic organization. Research activities within the Department are related to four core subjects within the discipline: business history, cooperative organizations, business development and entrepreneurship, and finally studies of industries with a special focus on the electricity industry. He is also the dynamic chairman of the nonprofit organization Stiftelsen Holistisk Medisin Scandinavia, which aims to support the scientific development, research, and documentation of complementary and holistic medicine in Scandinavia. E-mail: niels.j.andersen@bi.no. Website: www.bi.no/users/fgl93013/

**Joav Merrick, MD, DMSc,** is Professor of Child Health and Human Development affiliated with the Zusman Child Development Center and Division of Community Health at the Ben Gurion University, Beer-Sheva, Israel and presently the Medical Director of the Division for Mental Retardation, Ministry of Social Affairs, Jerusalem and the Director of the National Institute of Child Health and Human Development. He has numerous publications in the field of child and human development, rehabilitation, intellectual disability, disability, health, welfare, abuse, advocacy and prevention. Dr. Merrick received the Peter Sabroe Child Award for outstanding work on behalf of Danish Children in 1985 and the International LEGO-Prize ("The Children's Nobel Prize") for an extraordinary contribution towards improvement in child welfare and well being in 1987. E-mail: jmerrick@internet-zahav.net. Website: www.nichd-israel.com
